# The use of appetite suppressants among health sciences undergraduate students in Southern Brazil

**DOI:** 10.1590/S1679-45082013000100009

**Published:** 2013

**Authors:** Carlos Zubaran, Rubia Lazzaretti

**Affiliations:** 1 School of Medicine, University of Western Sydney, Sydney, Australia; The Biological and Health Sciences Center, Universidade de Caxias do Sul, Caxias do Sul, RS, Brazil; Universidade de Caxias do Sul, Universidade de Caxias do Sul, The Biological and Health Sciences Center, Caxias do Sul, RS, Brazil; 2 The Biological and Health Sciences Center, Universidade de Caxias do Sul, Caxias do Sul, RS, Brazil

**Keywords:** Appetite suppressants, Obesity, Students, Brazil

## Abstract

**Objective:**

To investigate the prevalence of appetite suppressant use among health sciences students in Southern Brazil.

**Methods:**

Undergraduate students (n=300) from seven health science undergraduate courses of the Universidade de Caxias do Sul completed a questionnaire about the use of substances to suppress appetite.

**Results:**

A significant percentage (15%; n=45) of research participants used appetite suppressants at least once in their lives. The most commonly used substances were sympathomimetic stimulant drugs (5%), including amfepramone (3.3%) and fenproporex (1.7%). The lifetime use of appetite suppressants was more prevalent among Nursing (26.7%) and Nutrition (24.4%%) students. There was no reported use of appetite suppressants among medical students. The use of appetite suppressants was significantly more prevalent among women. The majority of those who used these substances did so under medical recommendation. Most of users took appetite suppressants for more than 3 months.

**Conclusion:**

Lifetime use of appetite suppressants was substantial, being sympathomimetic stimulant drugs the most commonly used agents. Students enrolled in Nursing and Nutrition courses presented a significantly higher prevalence of lifetime use of appetite suppressants.

## RESUMO

**Objetivo:**

Estabelecer a prevalência do uso de moderadores de apetite entre estudantes da área da saúde na Região Sul do Brasil.

**Métodos:**

Estudantes universitários (n=300) de sete cursos da área da saúde da Universidade de Caxias do Sul completaram um questionário sobre o uso de moderadores de apetite.

**Resultados:**

Uma significativa porcentagem da amostra (15%; n=45) usou moderadores de apetite pelo menos uma vez na vida. As substâncias mais comumente usadas foram drogas estimulantes (5%), incluindo amfepramona (3,3%) e fenproporex (1,7%). O uso de moderadores de apetite foi mais prevalente entre estudantes de Enfermagem (26,7%) e Nutrição (24,4%%). Não foi relatado o uso de moderadores de apetite entre estudantes de Medicina. O uso de anorexígenos foi significativamente mais prevalente entre mulheres. A maioria dos que usaram supressores de apetite o fez sob orientação médica, como também usou tais substâncias por mais de 3 meses.

**Conclusão:**

O uso durante a vida de moderadores de apetite foi significativo entre estudantes da área da saúde, especialmente entre os matriculados em Enfermagem e Nutrição. As drogas estimulantes simpatomiméticas foram os agentes mais comumente usados.

## INTRODUCTION

Obesity is one of the most common chronic disorders in industrialized societies. The impact of obesity in terms of public health has been widely recognized^(1)^. The medical community currently defines obesity as a disorder with a multifactorial pathogenesis that produces systematic lifestyle changes and, in the most severe cases, requires pharmacological treatment^(2)^. Several classes of medications are available in the pharmacotherapy of obesity^(3)^. In recent years, there has been a considerable increase in the nonmedical use and abuse of prescription medications for obesity^(4)^. The chronic use of appetite suppressants may be associated with a higher risk for substance use disorders^(5)^.

The abuse of appetite suppressant drugs has been frequently reported as problematic in developing countries^(6–8)^. A household survey conducted in 1999 in São Paulo, Brazil's largest city, revealed a lifetime prevalence of 0.9% for the use of appetite suppressing drugs^(9)^. Surveys conducted in 1996 and 2001 with undergraduate students from the University of São Paulo (USP) revealed significant increases in the lifetime use of amphetamines (from 4.8 to 9.0%), as well as in use within the last 12 months and within the last 30 days^(10)^.

The use of appetite suppressing drugs in Brazil is most prevalent among women, who is usually correlated with the culture of slimness as a symbol of beauty^(6)^. Evidence from studies conducted in different regions in Brazil confirms that women are the predominant consumers of psychoactive appetite suppressants^(6,7,11)^. Data collected in two major Brazilian cities, São Paulo and Brasília, revealed a significant prevalence of appetite suppressant use among women, mostly amphetamine-like compounds^(6)^. A general survey conducted with undergraduate Health Science students in the Northern region of Brazil revealed that amphetamines were among the most commonly used illicit substances, being amphetamine use significantly more prevalent among students from the highest socioeconomic classes (11.9%)^(12)^.

The use of “diet pills” is particularly noteworthy in Southern Brazil, where a cross-sectional study, revealed a prevalence of 1.3% for the use of appetite suppressant drugs among adults of both genders^(7)^. Results from this study also revealed that the majority of users (81%) had only used appetite suppressants as prescribed by their own doctors. A survey conducted in Porto Alegre, the capital of the southernmost State of Brazil, also revealed considerable use of pharmacological agents for the purpose of weight control, including laxatives (8.5%) and diuretics (2.8%)^(13)^. In a study conducted in Florianópolis, another Brazilian Southern State capital, female students were twice as likely to consume appetite suppressant drugs than their male counterparts^(14)^. Additional evidence obtained in Southern Brazil confirmed further that women tend to consume amphetamine-like drugs considerably more than men, and primarily for the purpose of suppressing appetite^(15)^.

Although amphetamine-like drugs are probably the most conspicuous appetite-suppressant agents, other substances are also commonly used for weight reduction purposes. However, there are limited investigations on the use of additional appetite suppressant drugs. The fact that the widespread use of amphetamine drugs in Brazil has expanded to affect women from all echelons of the local society suggests that the magnitude of appetite suppressant abuse may be even more significant.

## OBJECTIVE

To investigate the use of different types of appetite suppressants in a sample of 300 students enrolled in Health Science majors at the Universidade de Caxias do Sul (UCS) in Southern Brazil.

## METHODS

### Sample recruitment

This study investigated a convenience sample of 300 students enrolled in the first semester of seven Health Science majors at the UCS in Southern Brazil as follows: Biology, Medicine, Nursing, Nutrition, Pharmacy, Physiotherapy and Physical Education. Participants attended classes according to a schedule obtained from the UCS academic office. The main inclusion criterion required research participants to be enrolled in the first semester of a higher degree course of the Health Sciences area at the UCS. All participants had to have Portuguese as their mother tongue.

### Data collection

All first term classes of the selected courses were attended by a member of the research team. Students were then informed about the purpose of the research during a brief structured announcement at the beginning of classes. After presenting the research objective in classes, research workers offered a questionnaire for completion for those willing to participate in the study. Students were informed that all research information would be treated as confidential, and participants could decline participation at any stage of the process. Questionnaires with consent forms were subsequently offered to those willing to participate in the study. Research participants completed the questionnaires without any form of assistance in the classroom. After completing the answers, which usually required from 10 to 15 minutes, all questionnaires and respective consent forms were collected by the research worker. Students who did not volunteer to participate in the study either remained in the classroom or briefly left the class. Data collection was concluded after 3 months of investigation.

### Informed Consent

This study was approved by the Ethics and Research Committee of UCS (approved research protocol #41). Research procedures including data collection, statistical analyses and interpretation of findings were conducted from 2004 to 2009. All volunteers signed a consent form in which the procedures involved in this project were described. All participants completed the questionnaires under minimal guidance by trained examiners, who followed standardized procedures.

### Instruments

Data were collected via a structured questionnaire, which included 24 items that investigated an array of factors related to the lifetime use of appetite suppressants. A list of allopathic and alternative medicines included stimulants, selective serotonin reuptake inhibitors (SSRIs), benzodiazepines, laxatives, hypnotics, lipotropic hormone, thermogenic substances (which contain L-carnitine, L-tyrosine and L-phenylalanine), herbal extracts and “others”. Specific questions explored the type of substance used, while others questions addressed the frequency of use, occurrence of side effects and additional factors related to the substance use. This questionnaire used was previously validated in a comparative study in Brazil^(7)^. Demographic information, including gender and age, were also colleted.

### Data recording and statistical analysis

Data from the questionnaires were firstly checked for completeness and subsequently computerized and stored in a data bank organized in an Excel^®^ spreadsheet. Files were converted to Statistical Package for the Social Science (SPSS)^®^ format and subsequently analyzed. Analysis of demographic variables was performed in relation to age, gender and educational background. Analyses of frequency of use and comparative analyses were also conducted. Whenever pertinent, two-way contingency tables using crosstabs were used to explore statistical relationships between two variables. These procedures comprised χ^2^ tests and analyses of Cramér's V contingency coefficient.

## RESULTS

### Demographic information

Approximately 75.3% (n=226) of the sample was female. The group's mean age was 26 years (range=17-43). Affiliations according to majors were as follows: Biology (8%; n=24), Medicine (8.33%; n=25), Nursing (16%; n=48), Nutrition (14%; n=42), Pharmacy (14.66%; n=44), Physical Education (25%; n=75) and Physiotherapy (14%; n=42).

### Use of appetite suppressants

Out of this sample, 15% (n=45) reported the use of appetite suppressants at least once in their lives. The prevalence of lifetime use medications containing either amfepramone or fenproporex was 4.66% (n=14), whereas 10.66% (n=31) of students used “other” substances with the purpose of reducing weight, including SSRIs (3%), plant extracts (1.66%), compounds classified as thermogenics (0.66%), benzodiazepines (0.33%), lipotropic hormone (0.33%) and other non-specified drugs (4.66%). Two participants reported using more than one appetite suppressant substance.

**Figure 1:**
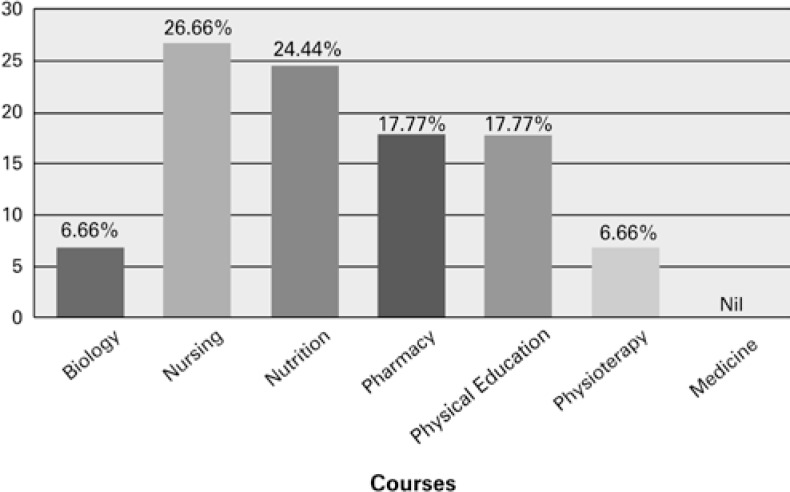
The percentage of use of appetite suppressants among undergraduate students in different Health Sciences courses

The lifetime prevalence of appetite suppressants in the total sample is demonstrated in figure 1: Nursing (26.66%); Nutrition (24.44%); Physical Education and Pharmacy (17.77%); Biology and Physiotherapy (6.66%); Medicine (nil). The majority of students who reported previous use of at least one appetite suppressant drug were female students (88.88%). A two-way contingency table analysis demonstrated a significant correlation between enrolment in specific undergraduate courses and the use of appetite suppressants, χ^2^ (6, n=300) =15.91, p=0.014, Cramér's V=0.23.

In terms of the source of advice for using appetite suppressants, 28.88% (n=13) of the students selfmedicated; 13.32% (n=6) mentioned a family member, a pharmacist, a friend or someone else; whereas 57.77% (n=26) of the sample received advice and prescription from a medical practitioner. Furthermore, almost a quarter of the research participants (24.44%) who took appetite suppressants visited a doctor during the preceding 6 months.

The prevalence of adverse reactions to appetite suppressants was noteworthy, with 10 to 20% of the volunteers suspending the use of these agents due to significant side effects such as irritability, nervousness, sleeplessness, sadness and emergence of physical signs and symptoms. Only 11.11% (n=5) of those who reported use of appetite suppressants continued to consult the prescribing doctor, whereas 46.67% (n=21) reported discontinuation of medical followup after using appetite suppressants. The remaining participants did not report any specific outcome. Two users (4.44%) reported increasing the dose of appetite suppressants without medical supervision, whereas 19 students (42.22%) did not indicate any answer related to dose increment. The mean length of time of appetite suppressant use was 99.48 days per user. Four respondents reported using appetite suppressants on a daily basis.

Furthermore, 48.88% (n=22) of those who reported use of appetite suppressants did so via a medical prescription, whereas 17.77% (n=8) purchased the medication in the pharmacy without a medical prescription. Eleven students reported “other” means of obtaining the medication.

## DISCUSSION

In this study, the use of appetite suppressant was reported among undergraduate students enrolled in the first semester of the Health Sciences courses at UCS. The results reported in this study demonstrate that the use of appetite suppressants was considerably prevalent in the sample investigated and that the use of appetite suppressants varied according to the type academic major of the user. The fact that some of the surveyed students will become health professionals with facilitated access to appetite suppressant substances in the future is of particular concern.

However, taking into consideration that these students were in the initial phases of their careers, it can be hypothesized that the significant acceptance of appetite suppressants, in a significant fraction of these students, was in fact related to their pre-academic views. The cross-sectional design of this study does not allow inferences regarding the possible attenuating effect of academic education on the use of appetite suppressants. It can also be hypothesized that students who followed careers in Health Science may have exposed themselves to appetite suppressants as a result of their preferential interest for health-related matters.

In this study, medical students were an exception to this trend, in that the prevalence of appetite suppressant use among them was significantly lower than in other Health Science's courses. These results contrast with previous evidence, which revealed significant levels of use of psychoactive drugs among medical students at UCS^(16)^. In contrast to the current investigation, no significant disproportion in gender distribution was found in the latter study, in wich students from the all years of the medicine were assessed. It is possible that methodological shortcomings may have prevented the detection of significant substance use among medical students. On the other hand, the absence of reported use of appetite suppressants among medical students may suggest that these future doctors may be less inclined to prescribe appetite suppressants for their patients, unless a tendency to prescribe these substances develops during their formative years in medical school. In the previously mentioned study, amphetamines were the only type of substance participants reported using prior to beginning medical training^(16)^, a finding that is consistent with additional evidence that demonstrates a more frequent use of psychotropic drugs during the concluding years of medical school^(17,18)^.

Most users of appetite suppressants in this study were women, which confirms previous findings that the use of appetite suppressant drugs is significantly prevalent among Brazilian women^(11,19)^. Brazilian female youngsters present great concern about their body image and slimness^(20)^. The widespread culture of slimness has been considered the main cause for an “epidemic” of unnecessary use of appetite suppressants^(11,19)^ in Brazil. An analysis of more than 40,000 medical prescriptions in São Paulo city revealed that such drugs were prescribed 10 times more frequently to women in comparison to men^(21)^. A previous study conducted in Brazil^(11)^ revealed that appetite suppressants were prescribed regardless of any indication of obesity. Similar results showed that 25.1% of persons using prescribed weight loss pills were below the minimum recommended body mass index, which indicated that these prescriptions were inappropriately used^(22)^.

The prescription of appetite suppressant drugs remains a common practice in Brazil^(10,11)^, particularly in Rio Grande do Sul, Brazil's southernmost state^(23)^. Most users of appetite suppressants followed medical advice when self-administering these medications. Yet, almost a third of the users of appetite suppressants took these medications without a medical assessment or prescription. More than a quarter of users reported purchasing appetite-suppressing agents directly from pharmacies, without any medical guidance. Inappropriate prescription of appetite suppressants has been reported in other countries^(8,24)^. The risks associated with the use of appetite suppressants have led some countries to adopt strict measures for the prescription of these substances^(25)^.

The maximum recommended length of time for therapy with centrally acting appetite suppressants was set at 3 months^(26,27)^. In the present study, the mean length of time of appetite suppressant use was almost 100 days, which exceeds the World Health Organization (WHO) recommended length of use. Most of the substances used by students in the current study present a stimulant effect on the human central nervous system, which is reflected in the most common adverse reactions reported by research volunteers, including restlessness, nervousness, irritability and insomnia. These adverse reactions may be so intolerable that many patients will cease medication use. In this sample, a significant proportion of users stopped using appetite suppressants due to adverse side effects. These untoward side effects may also include drug dependence, increased blood pressure, pulmonary hypertension, cardiac valvulopathy and eventually paranoid delusions^(28–31)^.

In Brazil many individuals attempted to lose weight by taking amphetamine-like drugs via compound formulas containing several substances^(11,19)^. These combinations of appetite suppressants, called “manipulation or magistral formulas”, used to be formulated in authorized pharmacies and contained combinations of amphetaminic drugs and benzodiazepines in addition to diuretics, thyroid agents, laxatives and medicinal plants^(7,11,19)^. In the current study, the use of compound formulas was not investigated. The fact that 17.77% of users of appetite suppressants obtained their drugs directly from the pharmacy, suggest that the use of formulas as mentioned above may have occurred in this sample as well.

The practice of dispensing combinations of amphetaminic drugs with other substances is widespread in Brazil^(32)^. In fact, the use of compounded diet pills has crossed geographical borders to Brazil, becoming also common among migrants in the United States. Results from an anonymous survey (n=307) conducted in one clinic and two churches with Brazilian immigrant women aged 18 to 50 living in the United States revealed that 18% of the clinic respondents and 9% of the church respondents reported using these diet pills^(33)^. Nearly two thirds of users of these pills reported adverse effects. Factors such as being unmarried, college educated, and having being advised by a North American physician to lose weight were associated with greater odds of imported diet pill use^(33)^.

In spite of the resolution passed by the Federal Medical Council (CFM) in Brazil^(34)^, which was ratified further by Ministry of Health CFM^(35)^, which prohibited the prescription of combinations of substances containing amphetaminic drugs, benzodiazepines, diuretics, hormone derivates, and laxatives, the dispensing of these combinations continued. Finally, in October 2011, the Brazilian National Agency for Sanitarian Vigilance (ANVISA) issued a resolution that prohibited prescribing, supplying and dispensing of anfepramone, mazindol and fenproporex^(36)^. Recent evidence suggest that after the enforcement of legislation mentioned above there was a progressive reduction in the dispensing of banned appetite suppressants in Brazil^(37)^.

This study presented some possible limitations. First, this investigation was developed only at a single university, which limits the possibility of results being generalized. Second, it is possible that many students who consumed appetite suppressants did not complete the survey honestly, which could compromise the results. A third possible limitation in this study has to do with the convenience sampling used in this study^(38)^. Convenience sampling may suffer from a number of *biases*, including the under-representation or overrepresentation of particular groups within the sample. This limitation may compromise generalization and interfere in the entire population, also compromising the external validity of the findings collected in this study. On the other hand, the choice for convenience sampling was based on several advantages, including the straightforward nature of the sampling procedure; its relatively reduced cost and time required its execution; as well as its usefulness to gather data and information that otherwise would not have been possible using the more systematized probability sampling techniques. Finally, considering that this study was conducted prior to the resolution issued by ANVISA in 2011, the results here presented may not accurately represent the current patterns of use of appetite suppressants in Brazil.

## CONCLUSION

In the present study, a concerning level of appetite suppressant use was observed among undergraduate students enrolled in first semester of Health Sciences courses at UCS. The results of this study confirmed that the use of appetite suppressant drugs was significantly more prevalent among women. The majority of those who reportedly used appetite suppressants followed the advice of a medical practitioner and many had to stop taking these medications due to unwanted side effects. The usual duration of treatment with appetite suppressants was superior to 3 months. Further comparative studies would be required in order to clarify if students enrolled in the Health Sciences majors present higher levels of appetite suppressant use than students from areas unrelated to the Health Sciences. Prospective studies would also help to establish if access to health-related information would, in contrast to what one would expect, facilitate the use of appetite suppressants or not. The findings presented in this study justify the need for further research in this area.
